# The Effect of Ivabradine on the Human Atrial Myocardial Contractility in an *In Vitro* Study

**DOI:** 10.1155/2019/7512318

**Published:** 2019-10-30

**Authors:** Ryan Chaban, Katja Buschmann, Anna Krausgrill, Andres Beiras-Fernandez, Christian-Friedrich Vahl

**Affiliations:** Department of Cardiothoracic and Vascular Surgery, The University Medical Center of Johannes Gutenberg University Mainz, 55131 Mainz, Germany

## Abstract

**Purpose:**

Ivabradine has emerged as a new antiarrhythmic agent that could compete with the traditional ones, such as *beta*-blockers. This experimental study aims to ascertain whether ivabradine directly interferes with the myocardial contractility in an *in vitro* environment.

**Methods:**

Myocardial tissues from the right atrial appendages of patients undergoing cardiac surgery were dissected to obtain 40 specimens from 20 patients (length: 3 mm), which were exposed to electrical impulses at a frequency of 75 bpm for 30 min to reach a steady state. Specimens were then categorised into four groups (each including five patients). The first group was the control, whereas the second, third, and fourth were treated with 60 nM, 200 nM, and 2 *μ*M ivabradine, respectively. We assessed five different contraction parameters before and after a 15 min treatment and calculated their relative changes, which were then compared to the control group.

**Results:**

Ivabradine has affected the force of contraction significantly *in vitro* (*p*=0.009). However, force of contraction decreased in both the control group (93.5 ± 4.7%) and the second group (94.1 ± 4.5%, *p*=0.8) and force of contraction remained unchanged in the third group (101.0 ± 4.1%, *p*=0.24) and increased significantly in the fourth group (108.9 ± 11.6%, *p*=0.008). There was no change in other contraction parameters, such as passive tension force (97.1 ± 5.1%, *p*=0.368), duration of contraction (99.1 ± 4.3%, *p*=0.816), time to peak (96.6 ± 3.0%, *p*=0.536), and time to relaxation (101.2 ± 7.0%, *p*=0.564).

**Conclusions:**

Ivabradine did not interfere with the contractile behaviour of human atrial tissue when it was used in therapeutic dosages in *vitro*. However, it increased the contractility slightly, when it was used in supratherapeutic dosage.

## 1. Introduction

Heart rate reduction (HRR), a cornerstone of the modern heart failure therapy [1–3], enhances the balance between the cardiac oxygen demand and supply by augmenting the coronary blood flow through a longer diastolic phase and decreasing the oxygen consumption owing to lowering the frequency. In addition, the heart rate decline leads to a better ventricular filling through an extended diastolic phase and an enhanced diastolic function because of better myocardial oxygen supply. Reportedly, in humans, as well as animals, the HRR enhances also the cardiac function in the long run by improving coronary collateralization [4–6].

Despite the acknowledged benefits of the HRR, its achievement remains complicated, as traditional antiarrhythmic agents still display their drawbacks. For example, *beta*-adrenoceptor blockers exert a negative inotropic effect and may cause undesirable side effects such as depression and lung function worsening [7–11]. In cardiac surgery, especially, a controlled reduction in the heart rate without interfering with the systolic cardiac function is needed frequently.

Ivabradine, an HCN (hyperpolarization-activated cyclic nucleotide-gated cation channel) blocker, was first approved for medical use by the European Medicines Agency in 2005 and by the United States Food and Drug Administration in 2015 and remains, to date, as the only clinically approved selective HCN-blocker. HCN-channels [12] underlie the “funny current” to induce the spontaneous depolarization of the pacemaker cells in the sinoatrial node, atrioventricular node, and Purkinje fibers [13–15]. By blocking these channels, ivabradine can exert a selective negative chronotropic effect upon the heart without interfering with the systolic function [16].

### 1.1. Aim of the Study

This study aims to investigate the effect of ivabradine on the cardiac contractility independently from the HRR. It used a well-established *in vitro* model to analyse the contractility of human atrial cardiac tissues in the presence and absence of ivabradine.

## 2. Materials and Methods

### 2.1. Ethical Approval

This study was conducted after obtaining clearance from the Ethics Board of Rhineland-Palatinate, Germany. We obtained individual written consent from patients for the use of disposed tissue arising from the surgical procedures, with the assurance of anonymity. No personal information was collected in this study.

### 2.2. Experimental Tissue and Preparation

The edges of the right atrial appendages that were routinely removed and discarded from patients undergoing cardiac surgery during the cardiopulmonary bypass were collected. Tissues were excluded in the presence of the following condition: age >90 or <18 years; severe cardiomyopathy, defined as an ejection fraction (EF) ≤30%; inflammatory or infective cardiac disease (e.g., endocarditis); congenital malformation; surgery for pathologies involving the right atrium (e.g., tricuspid regurgitation); digitalis therapy; and history of atrial fibrillation or flutter. Standard cardiovascular anesthesia was applied using total intravenous protocols with propofol and remifentanil. Noradrenaline, physiological solutions for volume substitution, and atropine were frequently used as required.

**Figure 1:**
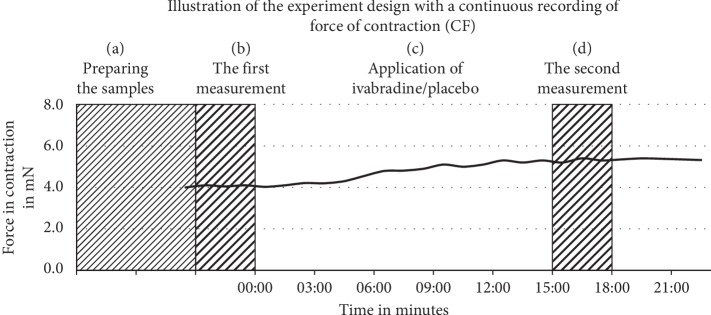
Experimental design. (a) Preparing the samples: specimens are left to stabilize and reach a steady state, before starting the experiment. (b) The first measurement: contraction parameters are recorded over a period of 3 min. (c) Application of ivabradine/placebo: ivabradine/placebo is applied over a period of 15 min during the continuous electrical stimulation. (d) The second measurement: contraction parameters are recorded again over a period of 3 min.

Samples were transported immediately after the surgical excision to the laboratory in a cold (4°C) modified Bretschneider's solution (prepared by the pharmacy of the University Medical Center of the Johannes Gutenberg University, Mainz, Germany), which contained 15 mM NaCl, 10 mM KCl, 4 mM MgCl.(H_2_O)_6_, 18 mM histidine.HCl.H_2_O, 180 mM histidine, 2 mM tryptophan, 30 mM mannitol, and 0.015 mM CaCl_2_.(H_2_O)_2_ and had a pH value of 7.2 (25°C). After that, trabeculae were manually prepared under the microscope to yield muscle specimens measuring about 3 × 0.5 × 0.6 mm^3^ (see Figure 1). Furthermore, these specimens were stored in dark cold (4°C) oxygenated Bretschneider's solution for 1–24 h, before being used in experiments.

### 2.3. Tissue Preparation

At the start of each experiment, every specimen was washed and warmed for approximately 10 minutes with Krebs–Henseleit buffer, which contained 118 mM NaCl, 25 mM NaHCO_3_, 4.6 mM KCl, 1.2 mM KH_2_PO_4_, 1.2 mM MgSO_4_, 1.3 mM CaCl_2_, and 11 mM glucose. Trabeculae were then mounted horizontally between two tweezers of the muscle investigation system (modified “Standard System for Muscle Investigation,” SH Heidelberg, Heidelberg, Germany) and exposed to a continuous flow of warm (35°C) Krebs–Henseleit buffer, gassed with a mix of 95% oxygen and 5% carbon dioxide at a rate of 0.5 ml/min, which kept the pH value at about 7.4. After a precise baseline length measurement, they were stretched to 110% of their slack length. Next, electrical stimulation was applied at a frequency of 75 bpm. The voltage was gradually increased from 1 V to a maximum of 10 V, until the maximal force of contraction (CF) of the specimen was reached. Thereafter, they were left to stabilize for 30 min to reach a steady state before starting the experiments.

### 2.4. Study Design

Four groups of experiments were conducted. The first group was kept in with Krebs–Henseleit buffer only (without ivabradine) and served as control. The second, the third, and the fourth groups were kept in 60 nM, 200 nM, and 2 *μ*M ivabradine, respectively. Each group included five different patients. Two experiments, using two samples, were studied from each patient, and the average was used to minimize the error.

Exposure to ivabradine/Krebs–Henseleit buffer lasted 15 minutes. Contraction parameters were measured twice: before applying ivabradine/Krebs–Henseleit buffer (para*X*_1_) and after (para*X*_2_). Each measurement lasted 3 minutes, and the average of the 3 × 75 bpm contractions was used for calculation. Then, the *relative change* in the contraction parameters (*X*_%_) was calculated according to the following equation:

(1) $$\begin{array}{c} \text{para}X_{\%}=100\times \text{para}X_{2}/\text{para}X_{1}. \end{array}$$

Figure 1 explains the design of this study.

**Figure 2:**
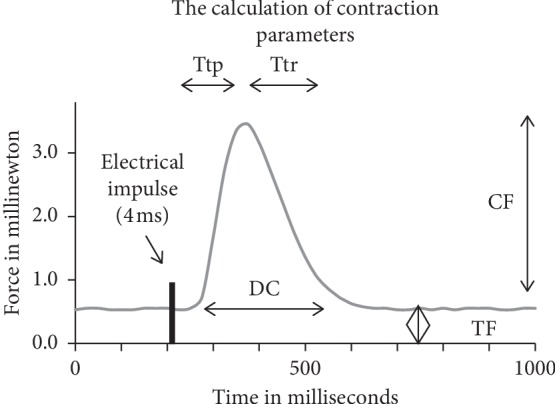
Calculation of contraction parameters. CF: force of contraction in millinewton; DC: duration of contraction in millisecond; TF: passive tension force in millinewton; Ttp: time to peak in millisecond; Ttr: time of relaxation in millisecond; ms: millisecond; mN: millinewton.

The following contraction parameters were measured: force of contraction in millinewton (CF), passive tension force in millinewton (TF), duration of contraction in millisecond (DC), time to peak tension in millisecond (Ttp), and time of relaxation in millisecond (Ttr).  Figure 2 explains how we calculated these parameters.

### 2.5. Source of Stock Solution of Ivabradine

We obtained ivabradine from Sigma-Aldrich, 3050 Spruce Street, St. Louis, MO 63103, USA, as *ivabradine hydrochloride powder*; this powder was used to prepare aqueous solutions in a concentration of 6 *μ*M, 20 *μ*M, and 200 *μ*M, which were then stored at −20°C. Next, we added these aqueous solutions directly to the Krebs–Henseleit buffer at a dilution of 1/100 to attain the required concentrations (60 nM, 200 nM, and 2 *μ*M) before conducting the experiments on the same day.

### 2.6. Data Acquisition and Statistical Analysis

Statistical analyses were performed using IBM-SPSS Statistics (version 23.0.0.0). Categorical variables were presented by frequencies and rates, and quantitative variables were described by their arithmetic means.

Owing to the versatility of the muscle specimens, they displayed wide differences in their baseline CF. As this study focused on the change in the contractility induced by the treatments rather than contractility itself, we analysed the *relative change* in the contraction parameters, instead of their absolute values. One-way analysis of variance (ANOVA) was then used, considering the “group” as an independent variable. The post hoc multiple comparison Dunnett's test was finally utilized to compare the three treatment groups against the control group. Notably, the Dunnett's test was two-tailed, and *α* = 0.05 was chosen for the significance level.

## 3. Results

A total of 40 experiments were conducted, in tissues from 20 patients (5 patients/group, average age: 63.7 ± 11 years).  Table 1 summarizes their patients' profile.

**Figure 3:**
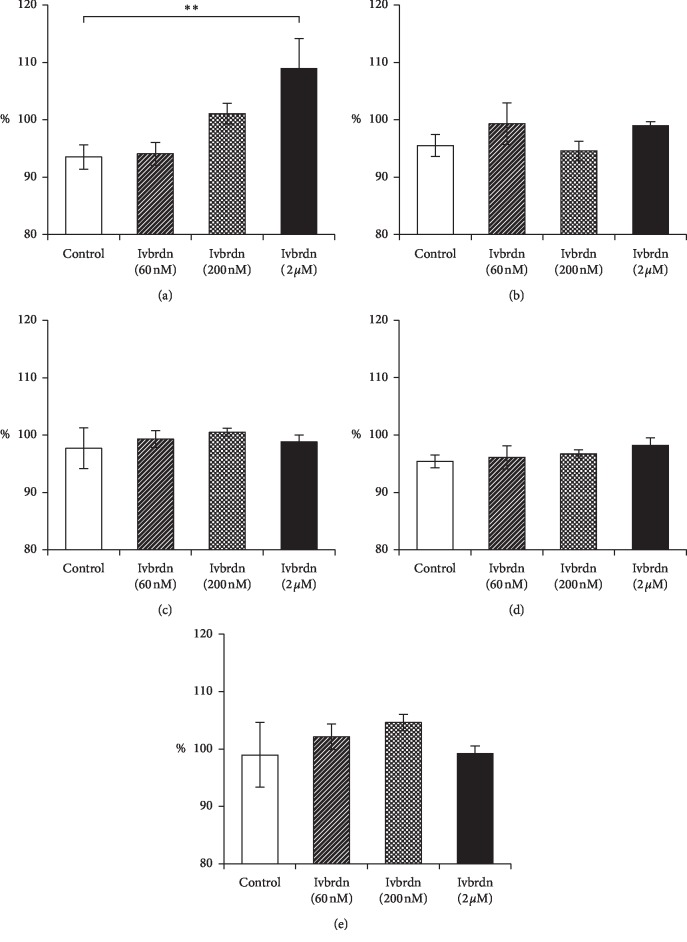
Measured contraction parameters and the averages with the standard errors of the mean. (a) Force of contraction. (b) Passive tension force. (c) Duration of contraction. (d) Time to peak. (e) Time of relaxation.

There was no change in other contraction parameters, such as passive tension force (97.1 ± 5.1%, *p*=0.368), duration of contraction (99.1 ± 4.3%, *p*=0.816), time to peak (96.6 ± 3.0%, *p*=0.536), and time to relaxation (101.2 ± 7.0%, *p*=0.564).  Table 2 and Figure 3 show these results.

## 4. Discussion

This study confirms the lack of a relevant inotropic effect of ivabradine, when it is used in therapeutic concentration. At a high concentration of 2 *μ*M, ivabradine exhibits a weak positive inotropic effect in this *in vitro* model.

Various effects of ivabradine on myocardial contractility have been reported before. Boldt et al. [17], for example, used an experimental setup similar to ours to assess the inotropic effects of ivabradine on both murine and human atrial cardiomyocytes; using a fixed contraction rate, they reported a concentration-dependent negative inotropic effect of ivabradine in 7 out of 10 subjects, while a concentration-dependent positive inotropic effect was observed in the remaining three subjects, as well as in murine cardiomyocytes. Both negative and positive inotropic effects in this trial were obtained in experimental concentrations between 10 and 100 *μ*M, which exceeded not only the concentrations used in clinical settings but also the maximum concentration in our trial. Furthermore, a negative inotropic effect resulting from application of ivabradine in concentrations above 10 *μ*M was reported by Pérez et al. in isolated guinea pig cardiac preparations [18]. Remarkably, Boldt et al. successfully blocked the positive inotropic effect of ivabradine by pretreating their samples with verapamil [17], suggesting an interaction between ivabradine and *L-type* calcium channels. Likewise, this effect is also reported by Bois et al., who demonstrated that ivabradine would block HCN channels at a concentration of 2 *μ*M, whereas it would block *L-type* calcium channels and the delayed outward potassium current at concentrations exceeding 10 *μ*M [16]. As such, it explains the paradoxical results of Boldt et al. [17]. It is essential to keep in mind that the concentrations needed to attain these effects exceed by far those used in clinical practice. Hence, it can be asserted that ivabradine exerts no relevant effect on the human myocardial contractility if applied in standard doses.

It might seem inappropriate to obtain samples from cardiac surgery patients comprising elderly individuals with various cardiac morbidities, but these patients precisely represent the targeted group of patients for the HHR therapy, and therefore, the use of these samples is advantageous. In addition, a broad range of heterogeneity existed between the patients, as well as between the performances of their samples. To counter that, we performed several experiments, enrolled patients strictly based on the inclusion and exclusion criteria, repeated the measurements twice for each patient, and analysed the change in the contractility under ivabradine rather than the contractility itself. Using a fixed rate of 75 bpm, which we consider the therapeutic heart rate in most heart failure patients, could be deemed misleading in a study considering a chronotropic agent, but because this study primarily aims to identify any effect that ivabradine exhibits on myocardial contractility and because the HRR effect of ivabradine has already been comprehensively discussed in literature [15, 16, 19, 20], we limited the framework of this study to observing the contractility under a physiological frequency. There are known differences between atrial and ventricular myocardium such as an approximately 15% smaller atrial cell volume yielding higher surface-area-to-volume ratio; smaller amplitude of systolic Ca^2+^ transients; accelerated rates of decline of systolic Ca^2+^; more sarcoplasmic reticulum- (SR-) mediated Ca^2+^ uptake; higher SR Ca^2+^ content, and a higher density of mitochondria in the ventricles [21, 22]. However, both possess similar contractile apparatuses and receptors [23, 24]. Hence, it is legitimate to hypothesize that whatever effect ivabradine has on myocardial contractility would be present, probably to different extents, in both atrial and ventricular cardiomyocytes and using higher concentrations of ivabradine facilitates the detection. Administration of 10 mg ivabradine (therapeutic dosage) causes a maximum plasma concentration of almost 60 nM [25–29]; therefore, we applied this concentration in our first experimental setup. The two higher concentrations, 200 nM and 2 *μ*M, would unveil any subtle effects not detectable when using the therapeutic dosage.

## 5. Conclusions

This study emphasises the lack of inotropic effect of ivabradine on the myocardial contractility when administered in therapeutic dosage. Ivabradine exhibits a small inotropic effect when used in higher concentrations.

## Figures and Tables

**Table 1:** Summary of the medical profiles and medications of the patients.

Donor Nr.	Group	Age (years)	Gender	BMI (kg/m^2^)	Diseases	Cardiac function	Surgery	Medications
D 001	Control	63	m	23	CAD, AHT, DM	Normal	Isolated CABG	ASA, bisoprolol, amlodipine

D 002	Control	66	f	26	CAD, AHT, ND, gout	Normal	Isolated CABG	ASA, bisoprolol, furosemide, amlodipine, vitamin D

D 004	Control	64	f	39	CAD, AHT	Normal	Isolated CABG	ASA, clopidogrel, bisoprolol, simvastatin, furosemide, amlodipine

D 005	Control	59	f	24	CAD, AHT, ND	Normal	Isolated CABG	ASA, ramipril

D 006	Control	49	m	31	CAD, AHT, ND, psoriasis	Normal	Isolated CABG	ASA, bisoprolol, simvastatin, amlodipine

D 008	2 *μ*Mol	71	m	29	CAD, IBS	Normal	Isolated CABG	ASA, clopidogrel, simvastatin

D 009	2 *μ*Mol	60	f	22	CAD, AVS, PAD	Moderately reduced	CABG + AVR	ASA, clopidogrel, furosemide, amlodipine, ramipril

D 010	2 *μ*Mol	75	m	33	CAD, DM, ND	Normal	Isolated CABG	ASA, simvastatin, furosemide, metformin

D 011	2 *μ*Mol	69	f	28	CAD, AHT	Normal	Isolated CABG	ASA, clopidogrel, bisoprolol, simvastatin

D 012	2 *μ*Mol	74	f	36	CAD, DM	Normal	Isolated CABG	ASA, bisoprolol, metformin, amlodipine

D 013	200 nMol	67	m	31	CAD, AHT, DM, ND	Normal	Isolated CABG	clopidogrel, bisoprolol, furosemide, metformin, amlodipine, lorazepam

D 015	200 nMol	62	f	43	CAD, AHT, ND	Normal	Isolated CABG	ASA

D 021	200 nMol	69	m	26	CAD, AHT, ND, PAD	Normal	Isolated CABG	ASA, clopidogrel, simvastatin

D 022	200 nMol	55	m	34	CAD, AVS, MVI, AHT	Normal	CABG + AVR + MVR	ASA, bisoprolol, furosemide, vitamin d

D 023	200 nMol	27	m	24	AVS, ND	Normal	Isolated AVR	ASA

D 027	60 nMol	77	m	25	CAD, AHT, DM, dN	Normal	Isolated CABG	ASA

D 028	60 nMol	77	m	30	CAD, AHT, DM, ND, dN	Normal	Isolated CABG	ASA, metoprolol, ramipril

D 029	60 nMol	59	f	38	CAD, AHT, DM	Normal	Isolated CABG	ASA, bisoprolol, simvastatin, amlodipine

D 030	60 nMol	71	m	27	CAD, AVS, AHT, PAD	Normal	Isolated AVR	ASA, bisoprolol, amlodipine, phenprocoumon

D 031	60 nMol	65	f	34	CAD, MVI, AHT	Normal	Isolated CABG	ASA, bisoprolol, amlodipine

CAD: coronary artery disease, AHT: arterial hypertension, DM: diabetes mellitus, ND: nicotine dependency, IBS: irritable bowel syndrome, AVS: aortic valve stenosis, PAD: peripheral artery disease, MVI: mitral valve insufficiency, dN: diabetic nephropathy, ASA: acetylsalicylic acid. Ivabradine has affected the force of contraction significantly *in vitro* (*p*=0.009). However, force of contraction decreased in both the control group (93.5 ± 4.7%) and the second group (94.1 ± 4.5%, *p*=0.8), force of contraction remained unchanged in the third group (101.0 ± 4.1%, *p*=0.24), and force of contraction increased significantly in the fourth group (108.9 ± 11.6%, *p*=0.008).

**Table 2:** Contraction parameters.

	Number of trials	Force of contraction before the treatment (mN)	Force of contraction (%)	Passive tension force (%)	Duration of contraction (%)	Time to peak (%)	Time to relax (%)
G0: control	5 × 2	1.3 ± 0.6	93.5 ± 4.7	95.5 ± 4.3	97.7 ± 7.9	95.4 ± 2.5	99.0 ± 12.6
G1: ivabradine (60 nM)	5 × 2	1.2 ± 0.5	94.1 ± 4.5	99.3 ± 8.1	99.3 ± 3.3	96.1 ± 4.5	102.1 ± 5.0
G2: ivabradine (200 nM)	5 × 2	1.1 ± 0.2	101.0 ± 4.1	94.6 ± 3.8	100.5 ± 1.6	96.7 ± 1.6	104.6 ± 3.2
G3: ivabradine (2 *μ*M)	5 × 2	1.5 ± 0.9	108.9 ± 11.6^*∗*^	99.0 ± 1.6	98.8 ± 2.7	98.2 ± 2.9	99.2 ± 3.1
Total/average	20 × 2	1.3 ± 0.6	99.4 ± 9.1	97.1 ± 5.1	99.1 ± 4.3	96.6 ± 3.0	101.2 ± 7.0

The averages of the measured force of contraction (CF) before the treatment in all groups and with the relative changes all contraction parameters. ^*∗*^A statistical relevance. Values were reported as mean ± standard deviation.

## Data Availability

The datasets used during this study are available as a supplement.

## Supplementary Materials

Supplementary Materials The supplementary material, which the authors have uploaded, “Ivabradine_results_supplementary.xlsb” contains two sheets and imbedded VBA macros. The first sheet “input tables,” with the macros, is used to calculate the following values from the original registration of the experiments: RT, CF, CF, Ttp, and Ttr (refer to the manuscript for the explanation of these values). The second sheet “Results” contains the individual results of the experiments in a tabular form (row 11 to row 27). Below that are the average values for the patients in row 33 to row 49 (from every patient, two experiments were conducted and the average was calculated, for example: cells B11 and C11 contain the calculated RT-values of the two trails of the donor D 001, and the average is represented in cell B33). From each variable (RT, for example) there is a value before and after and a calculated ratio “%.” Rows 54 to 85 contain the same values in another form (which was finally used in the manuscript).
